# Shared and Task-Specific Muscle Synergies during Normal Walking and Slipping

**DOI:** 10.3389/fnhum.2017.00040

**Published:** 2017-02-06

**Authors:** Mohammad Moein Nazifi, Han Ul Yoon, Kurt Beschorner, Pilwon Hur

**Affiliations:** ^1^Human Rehabilitation Group, Department of Mechanical Engineering, Texas A&M UniversityCollege Station, TX, USA; ^2^Deparment of Bioengineering, University of PittsburghPittsburgh, PA, USA; ^3^Deparment of Industrial Engineering, University of Wisconsin-MilwaukeeMilwaukee, WI, USA

**Keywords:** motor control, muscle synergy, slip, gait, fall

## Abstract

Falling accidents are costly due to their prevalence in the workplace. Slipping has been known to be the main cause of falling. Understanding the motor response used to regain balance after slipping is crucial to developing intervention strategies for effective recovery. Interestingly, studies on spinalized animals and studies on animals subjected to electrical microstimulation have provided major evidence that the Central Nervous System (CNS) uses motor primitives, such as muscle synergies, to control motor tasks. Muscle synergies are thought to be a critical mechanism used by the CNS to control complex motor tasks by reducing the dimensional complexity of the system. Even though synergies have demonstrated potential for indicating how the body responds to balance perturbations by accounting for majority of the data set's variability, this concept has not been applied to slipping. To address this gap, data from 11 healthy young adults were collected and analyzed during both unperturbed walking and slipping. Applying an iterative non-negative matrix decomposition technique, four muscle synergies and the corresponding time-series activation coefficients were extracted. The synergies and the activation coefficients were then compared between baseline walking and slipping to determine shared vs. task-specific synergies. Correlation analyses found that among four synergies, two synergies were shared between normal walking and slipping. However, the other two synergies were task-specific. Both limbs were contributing to each of the four synergies, suggesting substantial inter-limb coordination during gait and slip. These findings stay consistent with previous unilateral studies that reported similar synergies between unperturbed and perturbed walking. Activation coefficients corresponding to the two shared synergies were similar between normal walking and slipping for the first 200 ms after heel contact and differed later in stance, suggesting the activation of muscle synergies in response to a slip. A muscle synergy approach would reveal the used sub-tasks during slipping, facilitating identification of impaired sub-tasks, and potentially leading to a purposeful rehabilitation based on damaged sub-functions.

## Introduction

About 30% of “fall on same level” injuries contributed to losing 31 or more workdays in 2009 (Bureau of Labor Statistics US Department of Labor, 2009). During 2012, occupational injuries related to slips, trips, and falls resulted in a direct cost of over $16 billion in the USA (Liberty Mutual Research Institute for Safety, 2014). Also, according to Layne and Pollack (2004), “fall on same level,” was primarily triggered by slip. Slipping, tripping, and stumbling were the main causes of 64% of all falls in the US (Courtney et al., 2001). Consequently, slipping was reported to be the main contributor to fall initiation (Courtney et al., 2001; Gao and Abeysekera, 2004; Di Pilla, 2009). Furthermore, injuries caused by slip, trips, and falls have increased by 10% from 2013 to 2014 (Bureau of Labor Statistics US Department of Labor, 2015). Considering the prevalence and the increasing trend of fall-related injuries coupled with slipping being the main cause of falling, understanding the slip recovery process is of paramount importance in fall prevention.

While slips can be classified into several types (e.g., non-hazardous/hazardous; Lockhart T. E. et al., 2003; Lockhart T. et al., 2003), researchers agree that in general, maintaining balance during slipping requires fast and appropriate corrective responses (Tang and Woollacott, 1998; Cham and Redfern, 2001; Marigold and Patla, 2002). There were also several studies in which muscle patterns during slipping was investigated. Qu et al. (2012) found relations between muscle activation patterns and successful slip recoveries as well as failed slip recoveries. Studies also examined the latencies and the role of the muscle activation patterns of both lower and upper extremities on the recovery from a slip, implying intralimb, and interlimb coordination strategies in maintaining balance (Marigold and Patla, 2002; Marigold et al., 2003; Moyer et al., 2009). Also, studies focused on the stance leg after a slip found a minimum latency of 175 ms in muscle activations in major leg muscles (Chambers and Cham, 2007), and a 200 ms latency for restorative moments (Cham and Redfern, 2001).

Studies suggest that the CNS might control muscles using a low-dimensional organization of co-activated muscles, or *muscle synergy* (d'Avella et al., 2003; Ting and Macpherson, 2005; d'Avella and Bizzi, 2005; Overduin et al., 2012). In other words, muscle synergies are a group of co-active muscles recruited by a single control input, or *activation coefficient* (Ting and Macpherson, 2005; d'Avella and Bizzi, 2005). While the concept of the muscle synergies stays consistent among different researchers, they have tried different mathematical notations and terminologies to describe them. Some studies have used a vector with constant ratios for each muscle to present muscle synergies and a constant activation pattern for each synergy (Ting and Macpherson, 2005; Neptune et al., 2009), while other studies used time-varying muscle synergies and an activation pattern with an adjustable time delay (d'Avella et al., 2003; d'Avella and Bizzi, 2005). Not only have scientists different views about mathematical notations of synergies, but also they have different opinions about existence of such a lower dimensional modular organization to control and/or describe motor-tasks (Tresch and Jarc, 2009; de Rugy et al., 2013). Thus, a significant number of studies aimed to examine the muscle synergy hypothesis. Proponents of the synergy hypothesis as a descriptive tool have shown that synergies can be efficient in explaining the variability observed in the EMG signals for a vast range of activities in different animals; such as human gait, hand posture of macaques, posture of cats, and kicking in frogs (d'Avella et al., 2003; Ting and Macpherson, 2005; Neptune et al., 2009; Overduin et al., 2012). Muscle synergy as a neural control mechanism has been substantiated by several studies showing that electrical miscrostimulation on different parts of the CNS results in multi-degree-of-freedom motor behaviors and invariant postures, which may indicate presence of a coupling between the joint movements and muscle patterns (Zimmermann et al., 2011; Overduin et al., 2012; Steele et al., 2015). On the contrary, the opponents of this hypothesis state that the CNS is more likely to use an uncontrolled manifold or an optimal control schema to perform and control the motor-tasks (Todorov and Jordan, 2002; Valero-Cuevas et al., 2009) based on their experiments. Although, the opponents raised deep questions about muscle synergies with their research, other studies have shown that the uncontrolled manifold and optimal control methods often result in extraction of structures that are highly similar to muscle synergies (Krishnamoorthy et al., 2004; Todorov, 2004; Danna-Dos-Santos et al., 2009; de Rugy et al., 2013). This fact makes both endorsers and adversaries agree that muscle synergies are at least an effective descriptive tools to explain the variations observed in different motor-tasks using a lower dimensional organization. As a result, the intralimb and interlimb coordination happening in response to slipping incidents (Marigold et al., 2003; Moyer et al., 2009) may be better represented using muscle synergies. Muscle activation patterns during normal walking have also been described by muscle synergies. Interestingly, studies showed each of the walking muscle synergies correspond to a known sub-function of the gait cycle (e.g., propulsion, etc.; Clark et al., 2008; Neptune et al., 2009).

Muscle synergies could potentially be shared across activities. Studies done on animals (e.g., frogs; d'Avella et al., 2003; d'Avella and Bizzi, 2005) suggested that a few synergies were being shared between walking, jumping, and swimming. As muscle synergies of a motor task correspond to its physical sub-tasks, having the same mechanical goals and sub-functions in different motor tasks may result in having the same structure of muscle combinations and ratios, or shared muscle synergies. In other words, if two different motor task include a common mechanical sub-task, it is likely for a common muscle synergy to appear in both of the synergy sets. On the contrary, a task-specific mechanical goal is more likely to be reflected in a task-specific muscle synergy, which will not appear in the synergies of any other motor task. For example, Chvatal and Ting (2013) found that there exist shared synergies between unperturbed and perturbed standing and walking as well as the other non-shared, task-specific synergies. Also, Martino et al. (2015) investigated the synergies of normal walking as well as unstable gait conditions such as walking on a slippery surface and studied similarities of those motor behaviors. However, to our knowledge, no studies have investigated synergies during slipping (as an exclusive motor-task) and their existence, nor have any comparisons been made between synergies of normal walking and slipping to date. Thus, a muscle-synergy perspective may provide insights into whether a modular control strategy (synergies) is being used in response to slips or not, and if so, how similar or dissimilar those synergies are compared to normal walking synergies. Interlimb coordination could also be studied by extracting muscle synergies of both legs during these motor tasks. Muscle synergies could be useful in diagnosis and rehabilitation process (Roh et al., 2013). As synergies could present a sub-task of the main motor-task, extraction of slipping muscle synergies helps determining the sub-tasks of slipping. Also, activation coefficients of muscle synergies are informative as they represent the timing of activation for muscle synergies, and the corresponding sub-tasks. For the case in which a shared synergy exists, similarities in activation coefficients of the shared synergy would indicate an identical mechanical goal and timing performed in both motor-tasks. Thus, a muscle-synergic approach could provide a foundation for a comparison between healthy subjects' synergies and those of patients who are unable to recover from slips, hastening the diagnosis of impaired synergy and subsequently, identifying the impaired sub-task in them. It also may result in design of more efficient therapeutic interventions and targeted motor rehabilitation specifically intended to recover the malfunctioning sub-task (Dipietro et al., 2007; Roh et al., 2013).

The objective of this study is to examine and compare muscle synergies and time-series activation coefficients for two conditions: normal walking and slipping. We hypothesize that there exist both shared and task-specific muscle synergies between the two conditions. The rationale underneath this hypothesis is that a person may use a modularized lower limbs' control strategy while recovering from a slip, and some of these modules might be similar to those of normal walking. Accordingly, we hypothesize that the activation coefficients of the corresponding shared synergies that represent the timing of activation for these synergies would be similar for both normal walking and slipping.

## Methods

### Subjects

Eleven healthy young adults (6 males and 5 females, and age range: 22–33 years) free of balance disorders participated in this study. Everyone whose age was outside of the range of 18–35 and who might have issues with normal walking, e.g., pregnant women, were excluded from the study. Subjects provided informed consent prior to participation in the study and the study was approved by the University of Wisconsin-Milwaukee Institutional Review Board.

### Measurements, experimental protocol, and data processing

Subjects were fitted with a safety harness and surface electromyography (EMG) electrodes (Trigno, Delsys, Natick, MA) for four bilateral muscles and were asked to walk on a floor with four force plates (BP400600, AMTI, Watertown, MA) embedded. Using force plates, the kinetic data was collected at 1000 Hz. Collected ground reaction forces were used later to detect heel contacts using visual techniques. EMG data was sampled at 1000 Hz from four muscles for each side: medial hamstring (MH), tibialis anterior (TA), rectus femoris (RF), and medial gastrocnemius (MG). High-pass filtering and wrapping electrodes are commonly practiced to remove the possible movement artifacts. In this study, the electrodes were secured and stabilized using extra bandages around the electrodes to avoid artifacts due to the movement of the electrodes. Since the EMG sensors were wireless, cables might not contribute to movement artifact.

**Figure 1:**
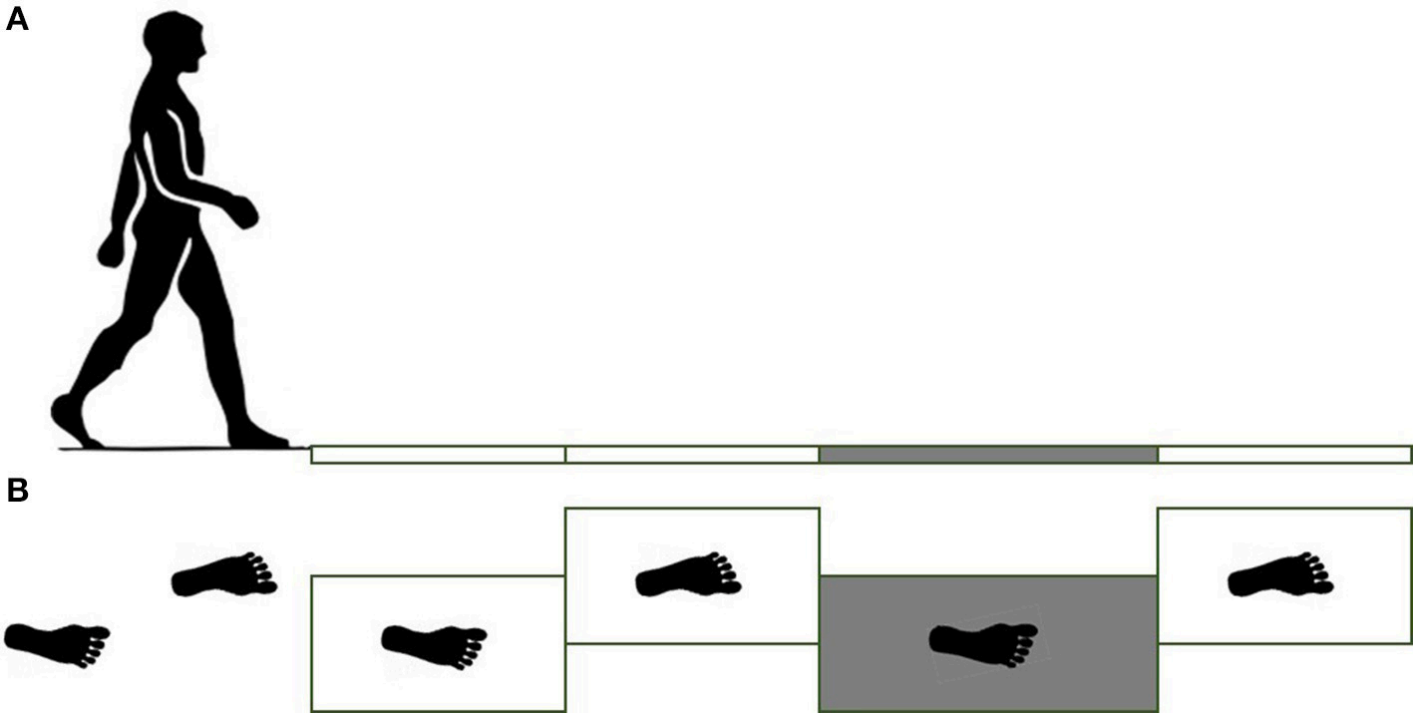
**Side view of force plates (A)** and top view **(B)**. For the slipping trials, the third force plate was contaminated by a diluted glycerol. Note right and left foot strikes.

The force plates were place in the middle of a 12 m long pathway in order to ensure that at least 5 steps were taken before stepping on the force plates. Subjects were asked to walk at their comfortable pace, step length, and cadence. Prior to the start of the gait trials, the subjects' starting position was adjusted to ensure that the subjects hit their right foot (leading foot, referred to as slipping/leading foot) on the third force plate (Figure 1). Subjects completed five unperturbed walking trials on the dry floor followed by one unexpected slip trial on the contaminant. During the slip trial, the third force plate (Figure 1) was contaminated to be slippery via applying a diluted glycerol (90% glycerol and 10% water) solution on it without informing the subjects. The third force plate was longer than the other force plates (0.4 × 0.8 m compared to 0.4 × 0.6 m) to minimize the risk that the subject would slip completely off of it during the slip (Figure 1).

**Figure 2:**
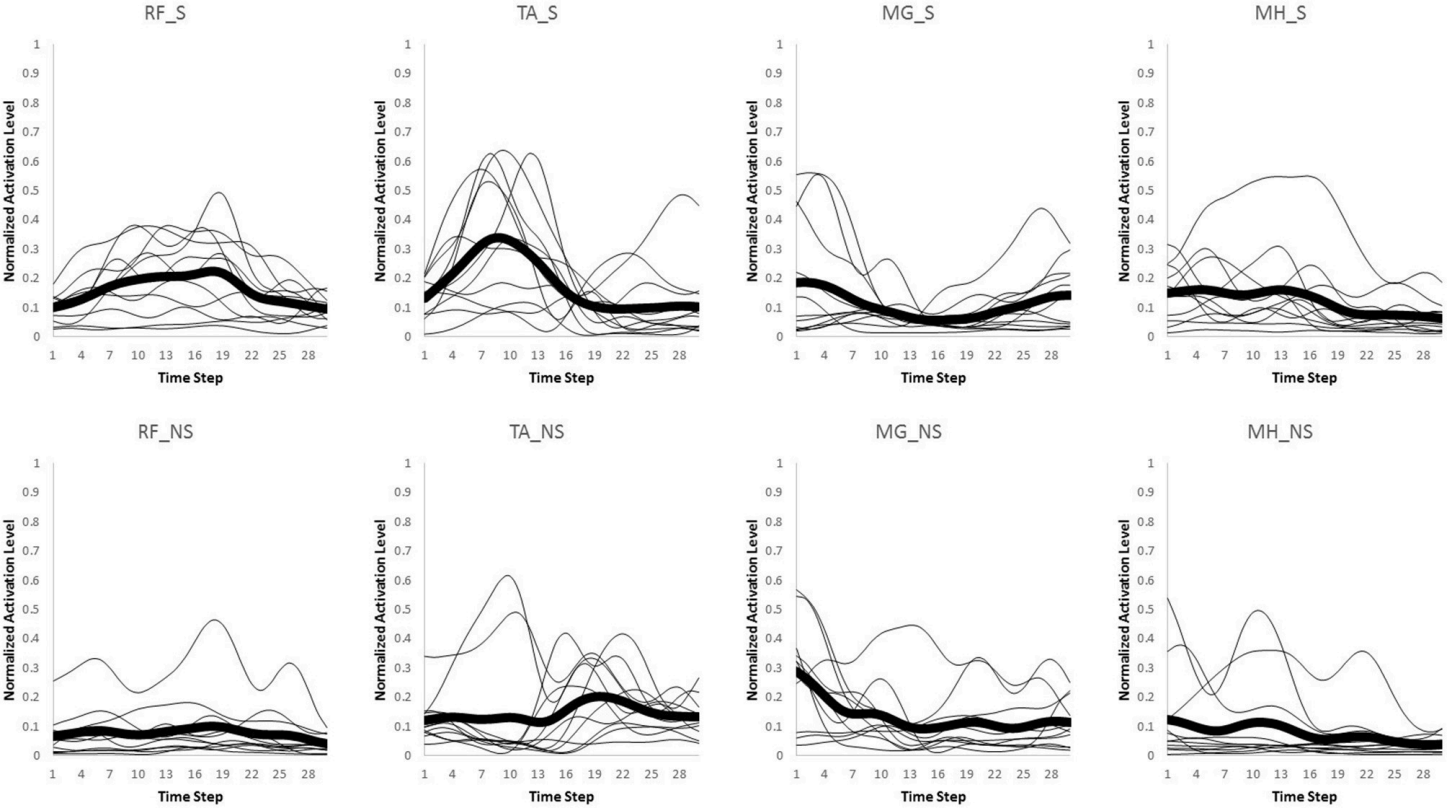
**Original muscle activation patterns during normal walking trials for the first 300 ms (integrated every 10 ms)**. The thick line represents the average value for every individual (thin lines).

**Figure 3:**
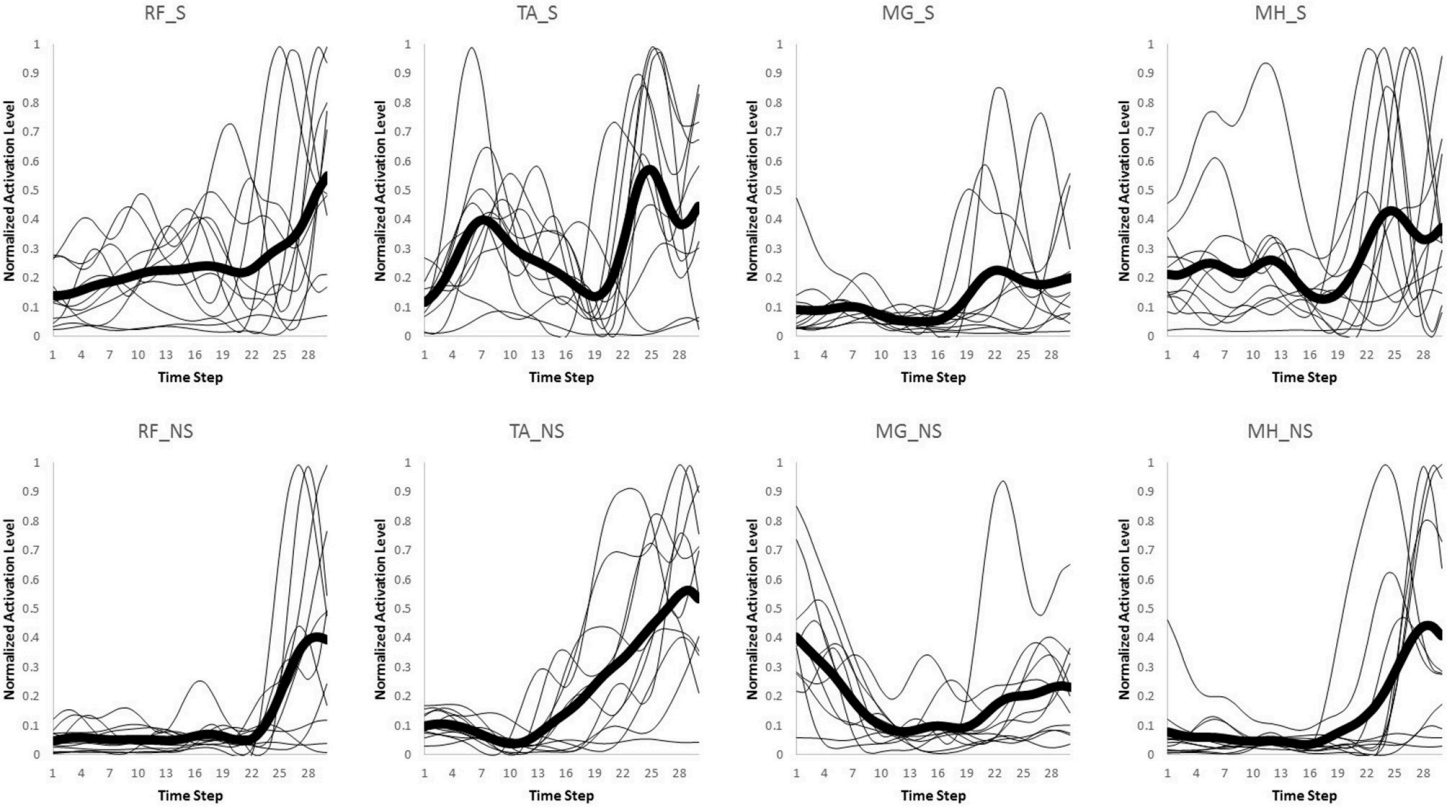
**Original muscle activation patterns during slipping trials for the first 300 ms (integrated every 10 ms)**. The thick line represents the average value for every individual (thin lines).

Only the first interval of 300 ms starting from heel strike on the third force plate was used for data analysis in both normal walking and slipping trials, since activation onset time of the EMG data for aforementioned muscles typically occur within the first 300 ms or 50% of stance after heel contact (Cham and Redfern, 2002; Marigold and Patla, 2002; Marigold et al., 2003; Moyer et al., 2009; Hur and Beschorner, 2012). The EMG activities for the eight muscles were recorded and processed via a full wave rectification and low-pass filtering (4th order Butterworth, cut-off frequency at 30 Hz) using MATLAB (v2014a, Mathworks, Natick, MA). Data were normalized to the maximum activation level among all trials within the same subject for each muscle. Finally, the data were integrated over every 10 ms interval, resulting in 30 data points for the whole 300 ms (Figures 2, 3). This interval was determined by d'Avella et al. (2003). As there existed several normal walking trials, the average of the all trials were used. Although, averaging trials may affect the variance-covariance structure of the data, in order to avoid having different time step size and timing at each data point, the trials were averaged. For each subject, the resulting processed EMG data were then assembled into a matrix, *M*, that had 30 rows corresponding to each time interval and eight columns corresponding to each muscle (i.e., *M* ∈ *R*^30 × 8^).

### Synergy extraction

Muscle synergy was considered to be a row vector, *w*_*i*_ ∈ *R*^1 × 8^, where each of the elements corresponded to each muscle's contribution to build that specific synergy. Also, time-series activation coefficient of the corresponding synergy was noted with a column vector, $c_{i}\in R^{30\times 1}$ with each element corresponding to a time step (= 30 in this study). Using the same iterative non-negative matrix decomposition algorithm introduced by Ting and Macpherson (2005) (via MATLAB functions *fmincon* and *lsqnonneg*), *n* muscle synergies ($W_{n\times 8}=\;\left[\begin{array}{c} w_{1}\\ \vdots \\ w_{n} \end{array}\right]$) and the corresponding *n* activation coefficients ($C_{30\times n}=\;\left[\begin{array}{ccc} c_{1} & \cdots & c_{n} \end{array}\right]$) were extracted for each subject's data during unperturbed walking condition and slipping condition, respectively. This algorithm identified the muscle synergies and time-series activation coefficients that best fit the resulting processed EMG data (*M*_30 × 8_). Note that *n* denotes the number of extracted synergies and can vary from 1 to 8 (= total number of muscles).

(1) $$M_{30\times 8}{}_{{}_{rebuilt}}=\sum_{i=1}^{n}c_{i}w_{i}=C_{30\times n}\times W_{n\times 8}$$

**Figure 4:**
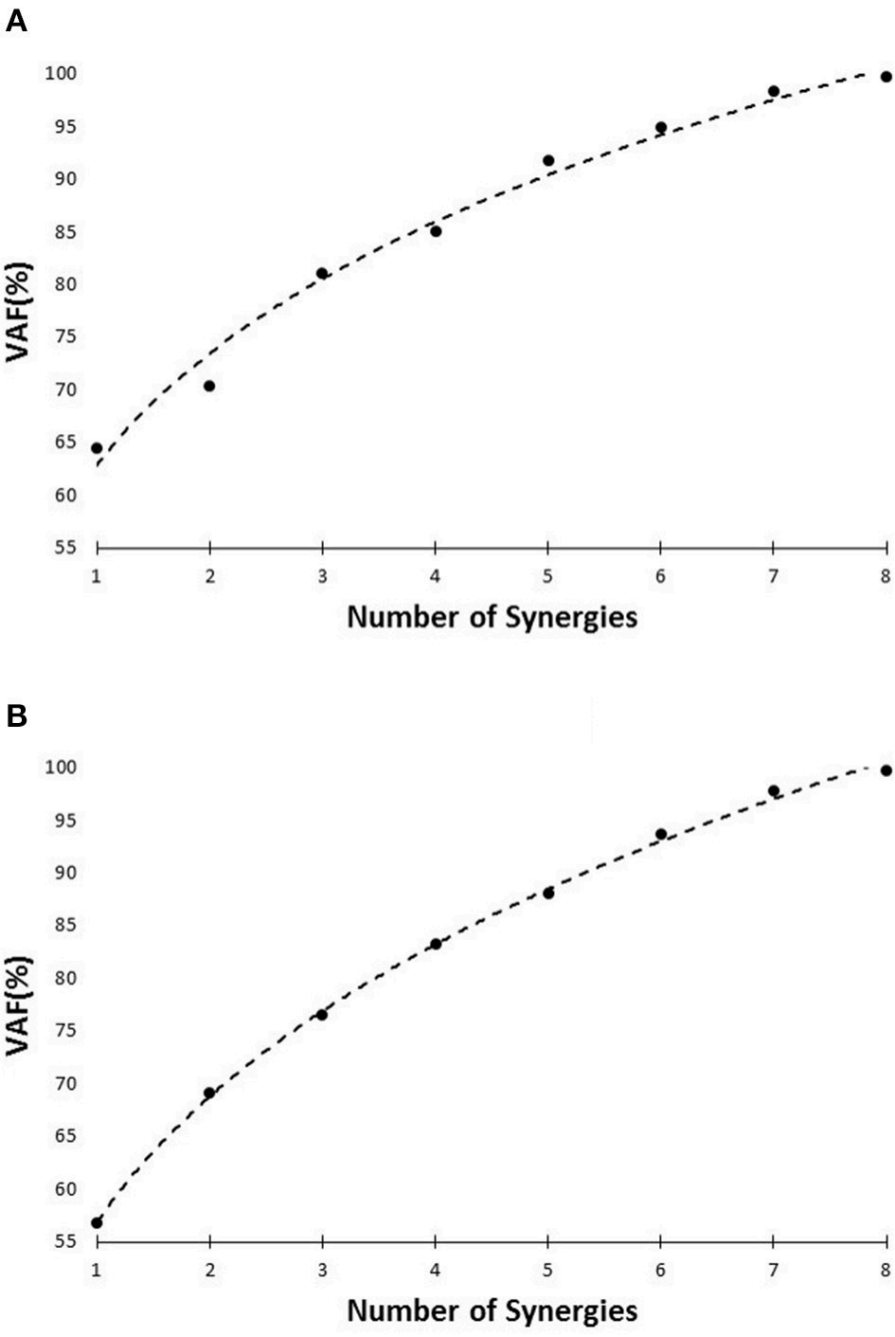
**VAF vs. number of synergies curve for slipping (A)**, and walking condition **(B)**, based on the pooled data set.

The number of synergies was chosen in a way to maximize the efficacy of the reproduced data using the lowest number of synergies possible. *Variability Accounted For* (VAF) was utilized as the metric (Ting and Macpherson, 2005; Neptune et al., 2009; Clark et al., 2010) to do so. VAF was defined (Equation 2) according to previous research (Neptune et al., 2009; Clark et al., 2010). The number of synergies was chosen using two criteria. The number of muscle synergies was the minimum of (1) The minimum number that could account for at least 75% of the variability of the data (Torres-Oviedo and Ting, 2010), and (2) at the minimum number at which adding an extra synergy did not contribute more than 5% in rebuilding the processed EMG data (Figure 4; Clark et al., 2010).

(2) $$VAF=1-\frac{||M_{30\;\times \;8,\;\;processed}-M_{30\times 8,\;\;rebuilt}|{|}_{F}{}^{2}}{||M_{30\times 8,\;\;processed}||F^{2}}$$

where the subscript *F* indicates the Frobenious norm.

### Reference subject selection and synergy ordering

**Figure 5:**
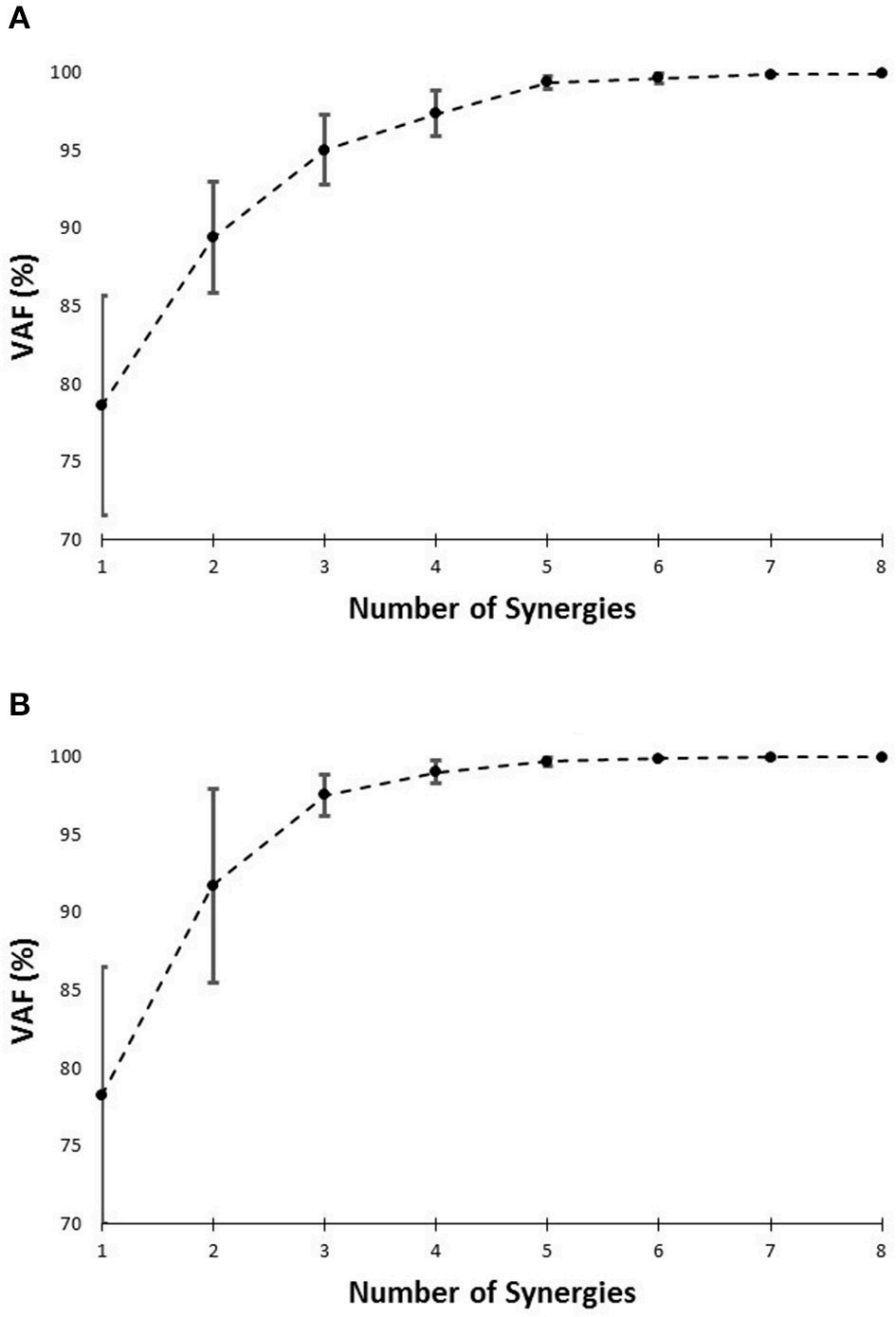
**VAF (averaged) vs. number of synergies curve for slipping (A)**, and normal walking condition **(B)**, based on each individual's data. Error bars indicate one standard deviation.

The synergies extracted using the abovementioned method would not have any pre-specified sequential order; meaning that a sorting is crucial to have all the similar synergies [i.e., with highest correlation (dot product) to each other, explained in details in the next paragraph] in the same order among subjects (Figure 5). Hence, once synergies were calculated for each individual, synergy referencing and ordering were performed to group similar synergies across subjects. Referencing and ordering were performed as follows: First, a reference subject was chosen for each condition (normal walking and slipping) whose synergies best described the synergies of all subjects (i.e., the subject whose synergies showed the maximum value of similarity to all other subjects' synergies). Similarity was quantified by uncentered correlation coefficients (d'Avella et al., 2003; Torres-Oviedo and Ting, 2010), ($r_{ij}=\cos\theta =\frac{w_{i}.w_{j}}{\left|w_{i}|.|w_{j}\right|}$ *for two given synergies*), for every possible pairs of synergies of a reference subject (i.e., subject *i*) and all the others subjects (i.e., subject *j, j*≠*i*; Torres-Oviedo and Ting, 2010; Roh et al., 2013). A pair of synergies was considered significantly similar if *r* > 0.7, and marginally similar if *r* > 0.45 (Torres-Oviedo and Ting, 2010). The reference subject was selected to be the one with the greatest number of significantly similar synergies (pairs with *r* > 0.7) with all the other subjects.

**Figure 6:**
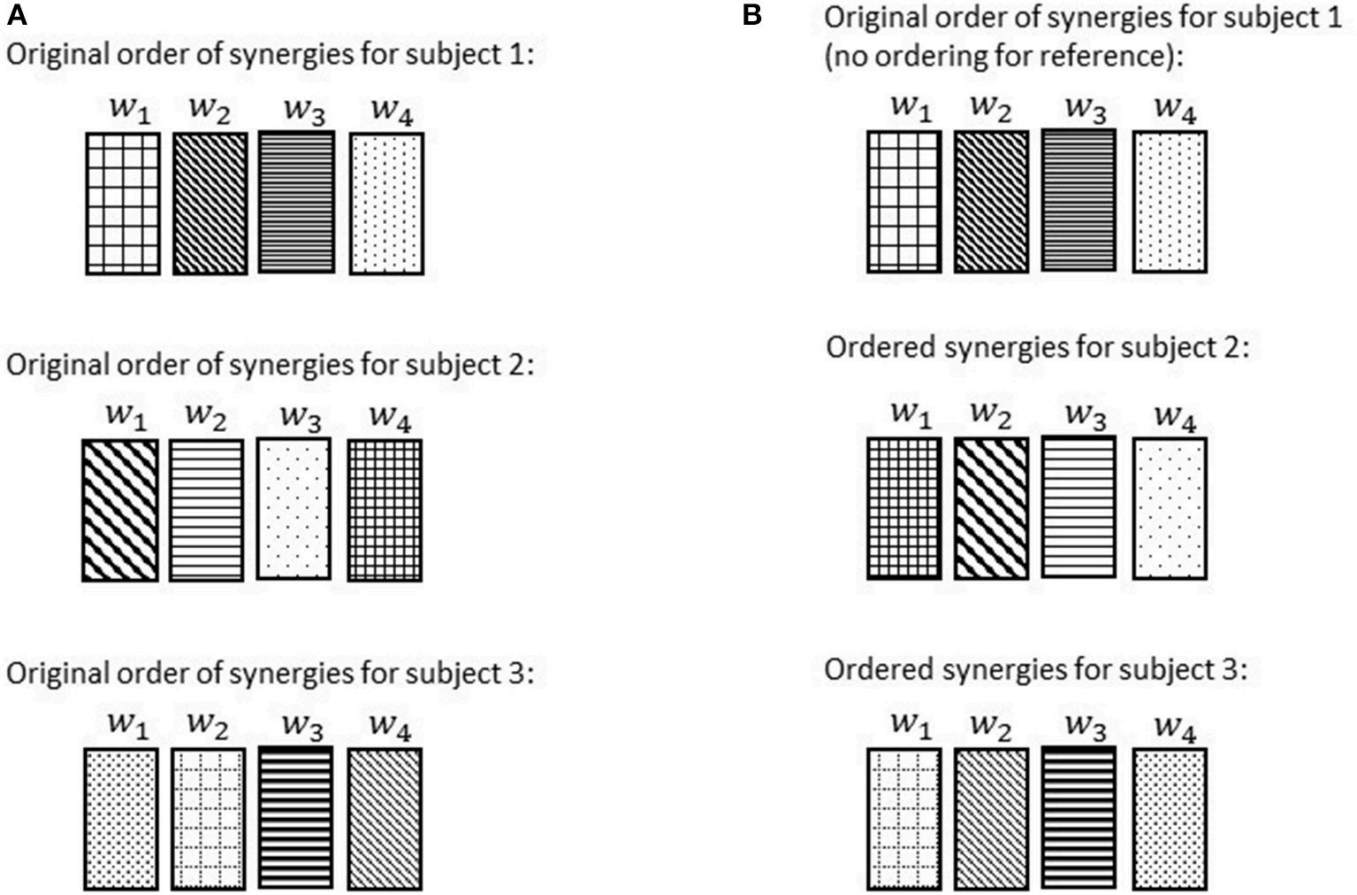
**Order of the normal walking synergies in different subjects before ordering (A)** and after choosing subject 1 as the reference and ordering the synergies accordingly **(B)**. Discrepancies of synergies are symbolized via hatch patterns. Note that after ordering, *w*_1_ for each subject would always refer to a synergy with the same hatch pattern (hatch pattern symbolizes characteristics).

Once the reference subject was determined, the ordered list of synergies, (*w*_1_, *w*_2_, _…_, *w*_*n*_), in the reference subject were changed such that the most common synergy comes the first. We chose the most common synergy as the one that significantly correlated with the maximum number of subjects (e.g., maximum number of *r* > 0.7). Once synergies were reordered in the reference subject, the orders of synergies of the other subjects were systematically modified as follows in order to match with the most similar synergies of the reference subject (d'Avella et al., 2003; Figure 6). The correlation *r* was computed for every possible pair of synergies between the reference subject and any other subject. Then, we picked the pair with highest similarity value, and synergies involved in that pair were removed from the set. Then, the highest similarity value among the remaining set was selected and again the pair was removed. This procedure was repeated until all the synergies were matched with their best matching pair. This step was performed so that similar synergies were in the same order across all subjects. In this way, the ordered list of synergies, (*w*_1_, *w*_2_, _…_, *w*_*n*_), would always present a unique set of synergies irrespective of the subject. Finally, the ordered lists of synergies were averaged across the subjects for presentation purpose (**Figures 8–11**).

### Investigation for shared and task-specific synergies between different gait conditions and their roles

**Figure 7:**
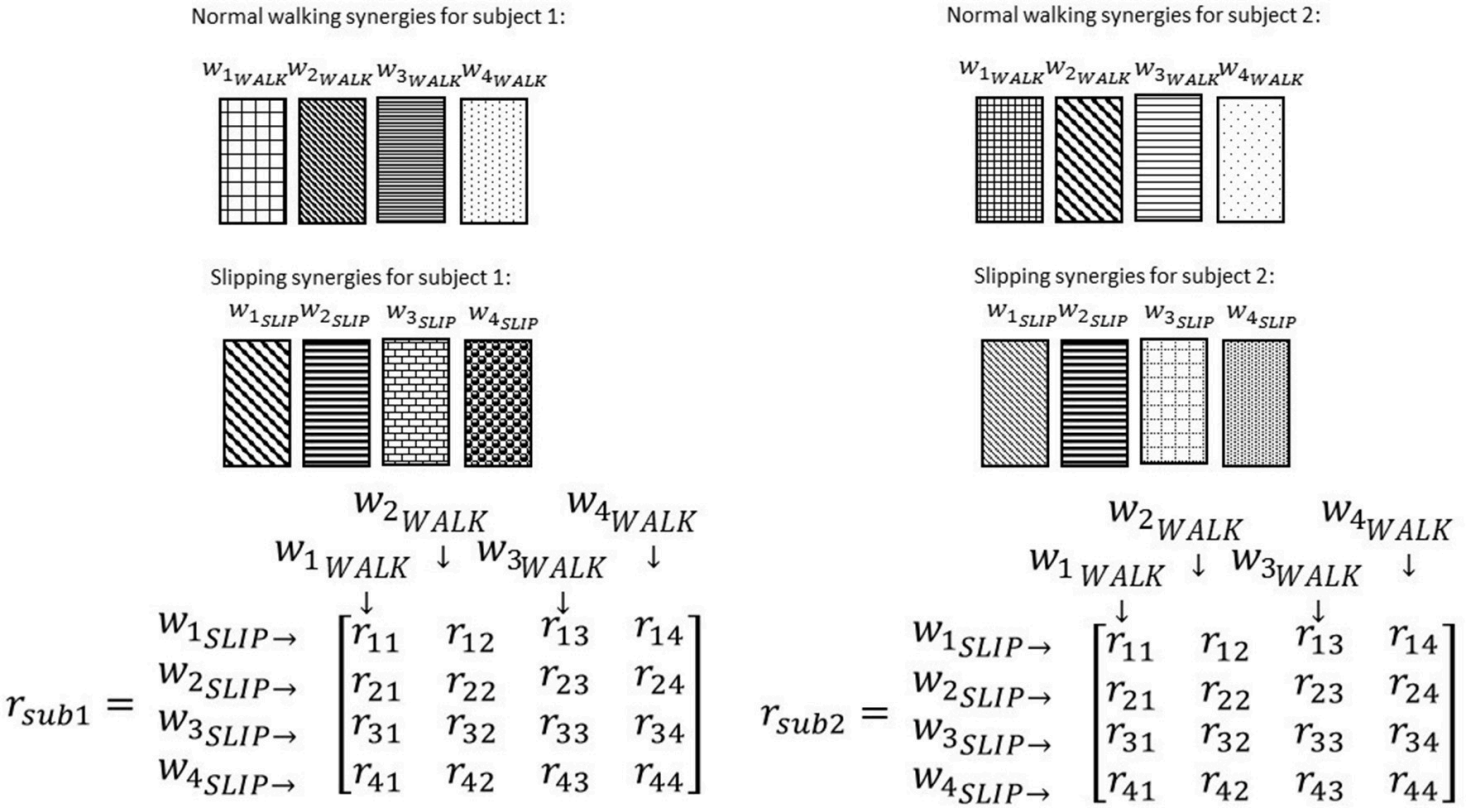
**Correlation coefficients are calculated after ordering the synergies according to a reference subject**. Note the same pattern and order in normal walking synergies (and slipping synergies) in different subjects. The intra-subject correlation of normal walk and slip synergies was determined via correlation coefficient matrix, *r*. Same elements of *r* matrix in different subjects always show the correlation of a specific pair of synergies.

To investigate if some synergies are shared between normal walking and slipping, normal walking synergies, and slipping synergies were compared for every individual. As before, uncentered correlation coefficients (*r*) were used to determine the similarity between the synergies of two tasks. For each subject, there were *n*^2^ possible pairs of synergies between normal waking and slipping conditions (Figure 7). For example, *r*_32_ represents the correlation between the third slip synergy and second normal walking synergy for all subjects. Once the correlation coefficient was calculated for each individual, one sample *t*-test (SPSS v21, IBM, Chicago, IL) was performed on the same *r*-value of all subjects to investigate if any of these pairs were significantly larger than the critical value across all subjects (*r*_*ij*_ > *r*_*critical*_, *p*_*value*_ < α). The significance level was fixed to be α = 0.05. The critical *r* values were set to be 0.7 and 0.45, respectively (Torres-Oviedo and Ting, 2010). The pairs of synergies that were correlated (either significantly or marginally) across all the subjects were considered *shared synergies* between two tasks, while the pairs that were not correlated were considered *task-specific synergies* (d'Avella et al., 2003). Gender effects were not included as a variable in the statistical analysis, since insufficient number of members in each group (6 members in males group vs. 5 in females group) would discredit the analysis.

This comparison method was repeated for time-series activation coefficients. However, activation coefficients were compared only for the shared synergies. The reason for this constraint was that for muscle synergies, if considered as building blocks of the nervous system, one can expect independent activation for each block (synergy) in general case. Thus, comparison of the similarity of activation patterns between different blocks (synergies) would not be meaningful, unless for the same blocks (the shared muscle synergies). We suspected that shared synergies have the same activation patterns since shared synergies are technically the same building blocks and might be activated with a similar pattern. However; we could not expect the similar activation patterns for task-specific synergies as they represent the activation of two totally different blocks. Additionally, since studies suggest a 200 ms latency for postural response to a slip (Cham and Redfern, 2001; Chambers and Cham, 2007), we also compared time-series of activation coefficients between two tasks for the first 200 ms.

At last, a simulation was run on each of the extracted synergies using OpenSim (SimTK, Stanford, CA) in order to observe their mechanical effect. The resulting muscle activations of every individual synergy was fed to a generic musculoskeletal system in OpenSim and the resulting movements were observed to conclude the role of each muscle synergy. Subsequently, based on the contribution weights of each muscle in the synergies, one could postulate the sub-task each synergy performs.

## Results

**Figure 8:**
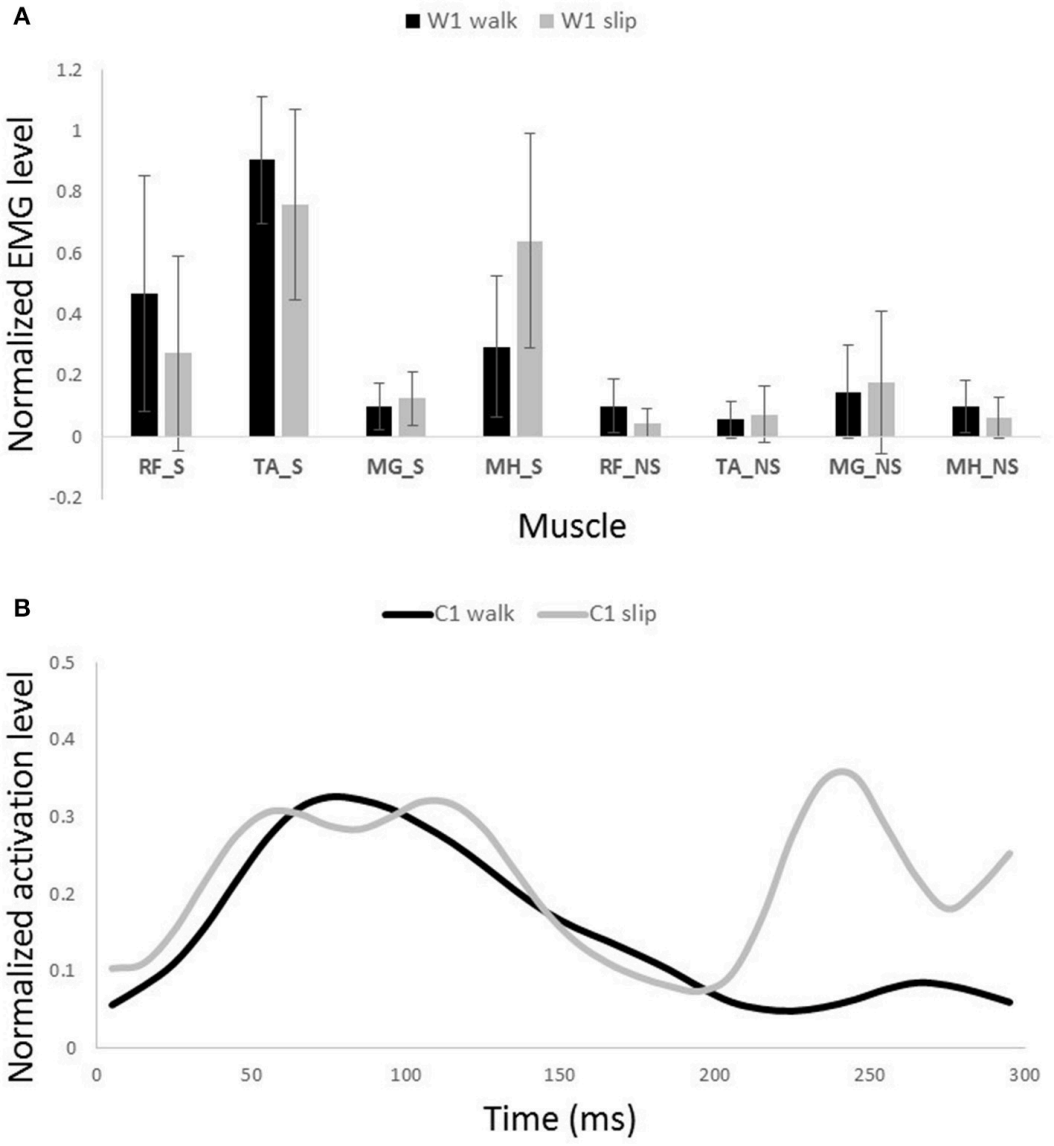
**Muscles synergies (A)** and their corresponding time-series activation coefficients **(B)** for the first shared muscle synergy between normal walking and slipping. Error bars indicate one standard deviation. Note that muscles belonging to slipping foot are shown by S while muscles of non-slipping foot are shown by NS.

**Figure 9:**
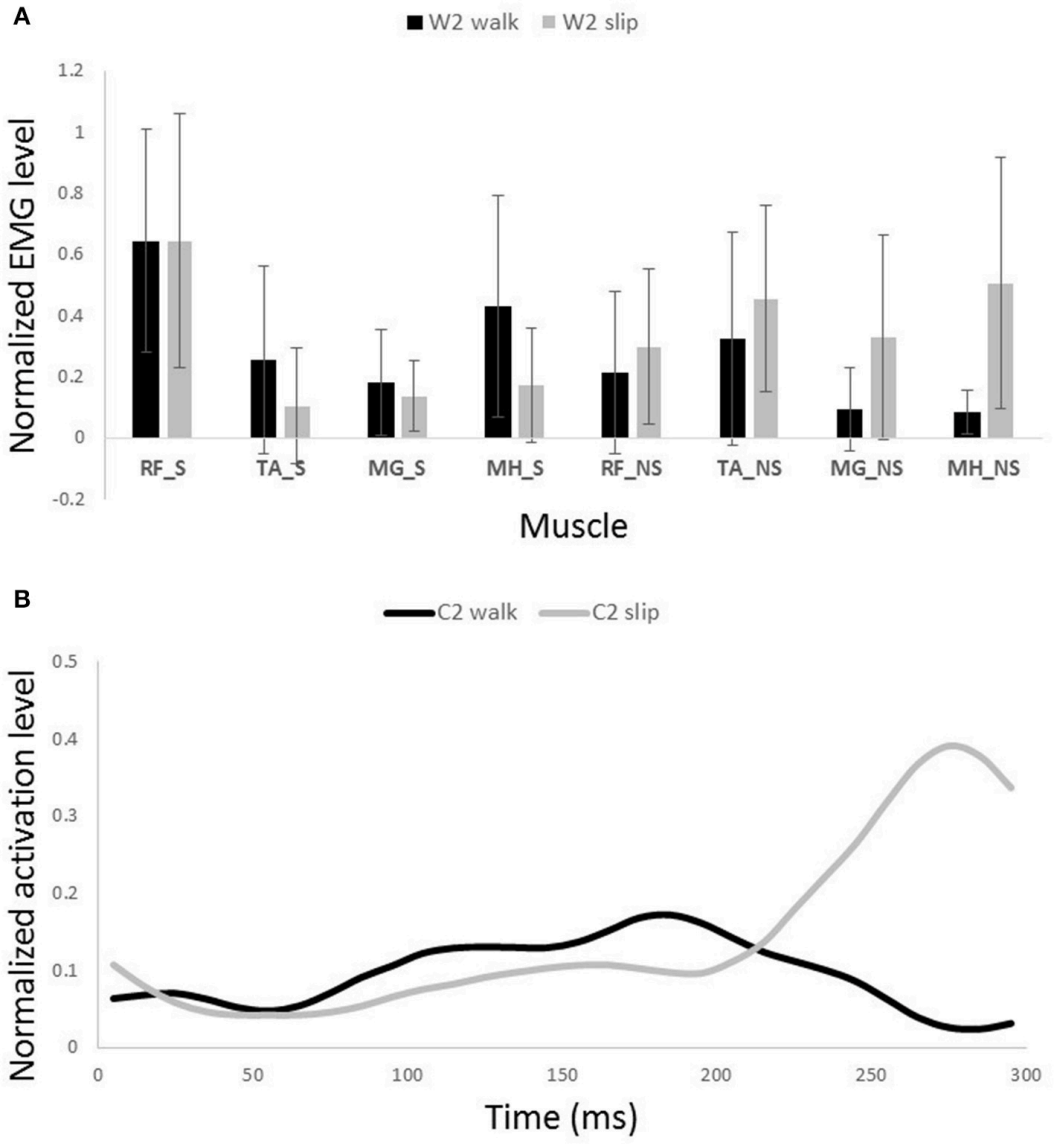
**Muscles synergies (A)** and their corresponding time-series activation coefficients **(B)** for the second shared muscle synergy between normal walking and slipping. Error bars indicate one standard deviation. Note that muscles belonging to slipping foot are shown by S while muscles of non-slipping foot are shown by NS.

**Figure 10:**
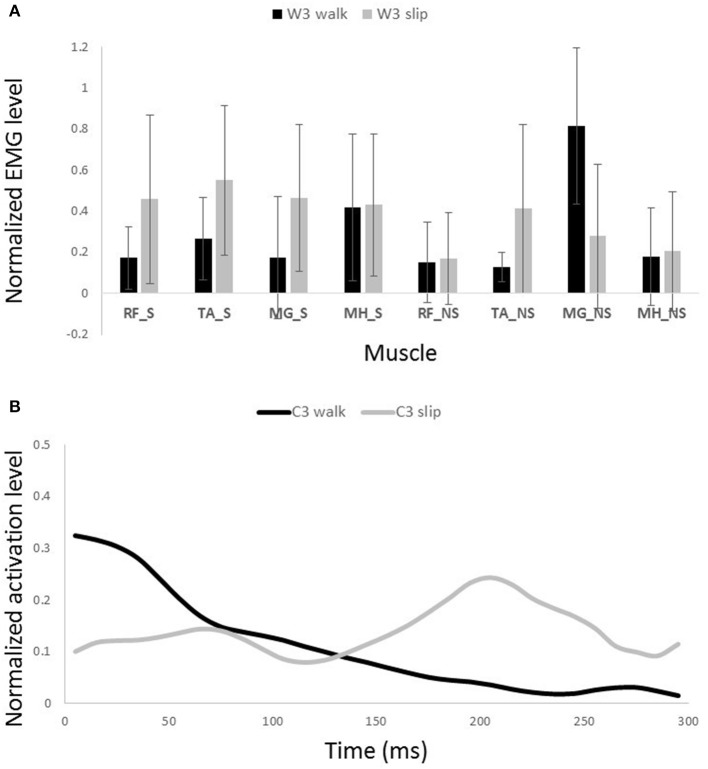
**Muscles synergies (A)** and their corresponding time-series activation coefficients **(B)** for the third muscle synergy of normal walking and slipping (considered task-specific). Error bars indicate one standard deviation. Note that muscles belonging to slipping foot are shown by S while muscles of non-slipping foot are shown by NS.

**Figure 11:**
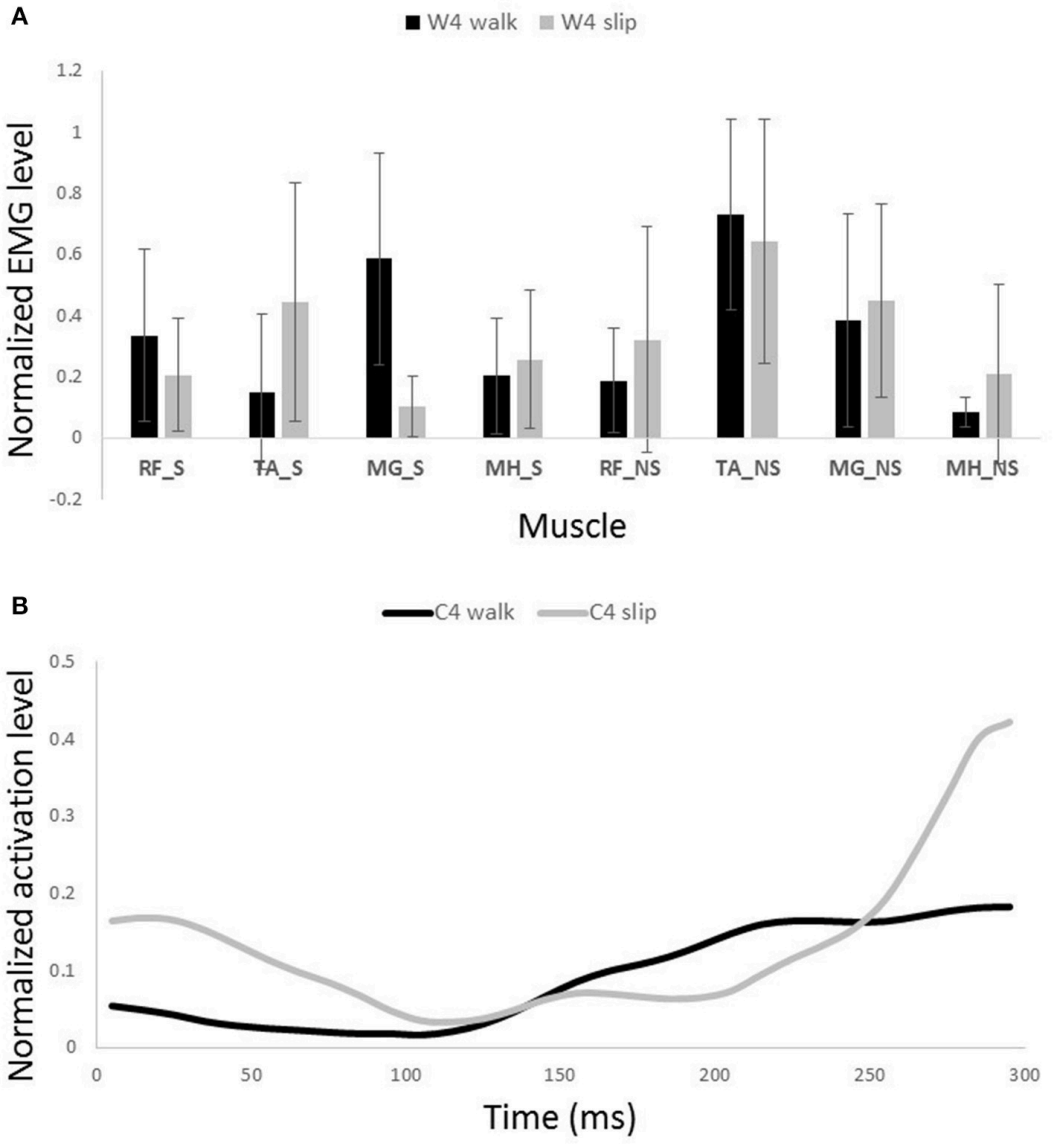
**Muscles synergies (A)** and their corresponding time-series activation coefficients **(B)** for the fourth muscle synergy of normal walking and slipping (considered task-specific). Error bars indicate one standard deviation. Note that muscles belonging to slipping foot are shown by S while muscles of non-slipping foot are shown by NS.

The setup effectively induced slip incidents on all subjects. The mean and the standard deviation for Peak Heel Velocity (PHV) during slipping and Slipping Distance were measured to be (0.90 ± 0.50 m/s) and (163.62 ± 101.89 mm), respectively. The EMG data was processed and prepared for synergy extraction. The original muscle activation patterns for walking and slipping are presented in Figures 2, 3, respectively. Using the aforementioned iterative non-negative matrix decomposition technique and varying the number of extracted synergies, corresponding VAFs were calculated for the pooled data from all subjects (Figure 4). Different research groups have used different values and techniques to find the thresholds for VAF (Torres-Oviedo and Ting, 2010; Chvatal et al., 2011; Roh et al., 2013). In this study, four synergies considered to be enough to account for variability of the normal walking and slipping data (Figure 4) as they successfully reconstructed more than 75% of the original *pooled* data (VAF ≥ 0.75) and also addition of an extra synergy did not contribute in reconstruction of more than 5% of the original data (Figure 4; Chvatal and Ting, 2013; Eskandari et al., 2016). The local VAF curves (for each individual's data) also substantiated our choice of four muscle synergies, accounting for more than 95%, for both walking and slipping condition (Figure 5; Ting and Macpherson, 2005). The number of synergies used in this study (four) also matches with the number of synergies used in similar studies with the same dimensionality (number of involved muscles) and motor task (walking and its sub-functions; Neptune et al., 2009; Clark et al., 2010; Roh et al., 2013). Subsequently, four normal walking synergies and four slip synergies were extracted (Figures 8–**11**). The corresponding time-series activation coefficients of those synergies were extracted as well.

One sample *t*-test results revealed that there are two pairs of synergies shared between normal walking and slipping. The first walking synergy was found *strongly* correlated with the first slipping synergy among subjects [*r*_11_ = 0.82 ± 0.13 > *r*_*strong correlation*_ = 0.7, *t*_(10)_ = 3.10, *p* < 0.01, Figure 8], while the second walking synergy was *marginally* correlated to the second slipping synergy [*r*_22_ = 0.62 ± 0.23 > *r*_*marginal correlation*_ = 0.45, *t*_(10)_ = 2.37, *p* = 0.02, Figure 9]. Hence, there are two pairs of shared synergies between normal walking and slipping. The other synergies were not correlated to each other, and these synergies were considered task-specific.

The complete 300 ms of activation coefficients of the shared synergies were tested to identify potential correlations. However, there was no strong correlation observed between the activation coefficients of the shared synergies. Only the activation coefficients of the first shared synergy showed a marginal correlation [*r* = 0.71 ± 0.18, *t*_(10)_ = 4.62, *p* < 0.001].

Interestingly, comparing the time-series of activation coefficients of the shared synergies for the first 200 ms revealed two significant similarities. The activation pattern of the first shared synergies were significantly correlated between normal walking and slipping conditions for the first 200 ms after heel contact [*r* = 0.84 ± 0.17, *t*_(10)_ = 2.72, *p* = 0.01, Figure 8B, first 200 ms]. The time courses of activation coefficients for the second shared synergy were also significantly correlated between normal walking and slipping conditions for the first 200 ms after heel contact [*r* = 0.59 ± 0.21, *t*_(10)_ = 2.25, *p* = 0.02, Figure 9B, first 200 ms].

## Discussion

Muscle activities for both lower limbs during gait and slipping were successfully presented. This study found four muscle synergies for each condition, of which two were shared between normal walking and slipping tasks, suggesting similarities between the required sub-tasks during normal walking and slipping tasks. As stated before, different research groups have used a wide range of VAF values and standards in their muscle synergy studies to decide number of synergies. This fact shows that there is no commonly accepted VAF threshold and one might simply find other criterions conservative or flexible. In this study, we tried to accommodate local, global, and “<5% growth” VAF conditions which are the most prevalent criteria introduced by different groups. Yet, other researchers may still prefer other values due to the existing uncertainties about this issue. Furthermore, limited number of muscle synergies (i.e., four synergies in comparison to eight muscles) used by the CNS during slipping shows the efficacy of muscle synergies in accounting for variation of a motor-task using a low dimensional modular organization, since the degrees of freedom are reduced to as low as four out of eight potentially available muscles in response to a slip. Finding of 6–7 synergies to control both walking and slipping stay consistent with the concept of synergies as a declarative and descriptive mean. Finally, only one of the time-series activation coefficients for the two shared synergies were correlated for the first 300 ms after heel contact. Interestingly however, both of the time-series activation coefficients for the two shared synergies were correlated during the first 200 ms after heel contact and deviated afterward according to the latencies and sub-functions reported for postural response to a slip (Cham and Redfern, 2001).

The synergies could have a specific functionality (Ting and Macpherson, 2005) and possibly could be interpreted as physical sub-tasks of the original motor behavior (d'Avella et al., 2003; Ting and Macpherson, 2005; Clark et al., 2008, 2010). Considering this fact along with the extracted muscle synergies of slipping and walking, one could postulate the sub-task each synergy performs based on the contribution weights of each muscle in the synergies.

The possible role of the first shared synergy was to decelerate the leading limb. This mechanical goal stays consistent with the known sub-tasks of the gait cycle at terminal swing phase. Pretibial and hamstring muscles group are known to be activated at the end of swing phase and in the early stance phase (Basmajian and De Luca, 1985; Medved, 2000; Rose and Gamble, 2005), in order to decelerate the leading limb and position the foot and arrange the contact. These sub-tasks are needed in both terminal swing phase and also in response to slips. The primary response to slip is to bring the slipping leg back near the body and shifting the COM forward (Cham and Redfern, 2001; Nazifi et al., 2015) which is possibly achieved by activation of this synergy. This common mechanical goal explains this synergy being shared between slipping and walking. The sub-tasks are produced by superpositioning the role of the main activated muscles in this synergy, namely TA_S and MH_S (Figure 8A). Also, a relatively high activation of RF_S was observed in this synergy. The co-activation of MH_S and RF_S would result in a stiffer knee joint on the slipping limb in order to avoid knee buckling while enduring the body weight. The simulation also verified the aforementioned role for this synergy in generating a hip extension as well as dorsiflexion on the leading leg. Considering the role of the hamstring in decelerating the lower limb (Rose and Gamble, 2005; Lockhart and Kim, 2006), activation of hamstring in early-stance phase results in deceleration of the slipping leg (Yang and Pai, 2010; Qu et al., 2012). Moreover, activation of TA_S causes a dorsiflexion to elevate anterior part of the leading foot and arrange the heel to strike. It also helps to store energy and prevents from foot drop or foot slapping phenomenon (Rose and Gamble, 2005).

The second shared synergy seemed to prepare the weight transfer to the leading limb. This sub-function happens at early stance phase of the gait and are mainly achieved by activation of the quadriceps muscles. As stated by Medved (2000), shortly after the heel strike, the quadriceps muscles group (RF in this study) contract in order to absorb the shock and provide more support to stabilize the knee and pelvis joint on the leading leg. This stabilization prepares the leading leg for weight transfer. Abovementioned sub-functions are also required in response to a slip. The secondary response to slip is to extend the knee and flex the hip of the slipping leg to avoid knee buckling and continue gait (Figure 9A; Cham and Redfern, 2001). Once again, the common mechanical goal substantiates this synergy being shared between the two conditions. Expectedly, the main activated muscle in this synergy was RF_S that contributes in knee extension, hip flexion, and weight acceptance. However, there was a slight difference between slipping and walking synergies (Figure 9A). A larger knee flexion angle was observed in the slipping synergy (most probably due to activation of MH_NS and MG_NS on the trailing foot) matching with previous studies. As the swing phase of the trailing limb is disturbed by the slip, these activations prevent the fall as the leading limb is not ready to accept body weight (Moyer et al., 2009). The simulation also resulted in knee extension and hip flexion on the leading foot, verifying the above mentioned arguments. Appearance of these muscle activations from both legs in a single synergy substantiates that the interlimb coordination in slip recovery might be rooted in synergies.

Other two synergies and their functionalities are considered task-specific. Although, having similar muscle synergies and activation between normal walking and slipping may seem to substantiate synergies as a neural control mechanism, having dissimilar synergies is more likely to support muscle synergies as a descriptive tool. That is because if synergies were a control mechanism, we would see identical synergies and activations during walking and the first 200 ms of slipping due to the reaction time. However, as W3 and W4 of walking are not used during early slipping (which is the same as walking), we claim that synergies are rather a descriptive tool instead of a neural control mechanism. Our findings show that the third normal walking synergy can be considered as the propulsion provider on the non-slipping leg according to the activation of MG_NS (Figure 10A). The simulation result also showed a high plantarflexion and knee flexion substantiating this expectation. This phasic sub-function happens at late stance of the trailing limb to provide the propulsive force and accelerate the body. However, as the terminal stance phase is interrupted during a slip, it seems cogent to lack this synergy during slipping. while the fourth synergy is responsible for dorsiflexion of the non-slipping foot to clear foot and avoid the toe from hitting the ground during swing and accomplishing foot flat when swing is terminated (Moyer et al., 2009; Figure 11A).

On the other hand, the third slipping synergy seems to stabilize and stiffen joints on both legs via activating almost all of antagonist muscles equally (Figure 10A). Moreover, the fourth slipping synergy contributes to dorsiflexion of the non-perturbed limb and might show the measure to avoid tripping during the slip (Marigold et al., 2003; Figure 11A).

**Figure 12:**
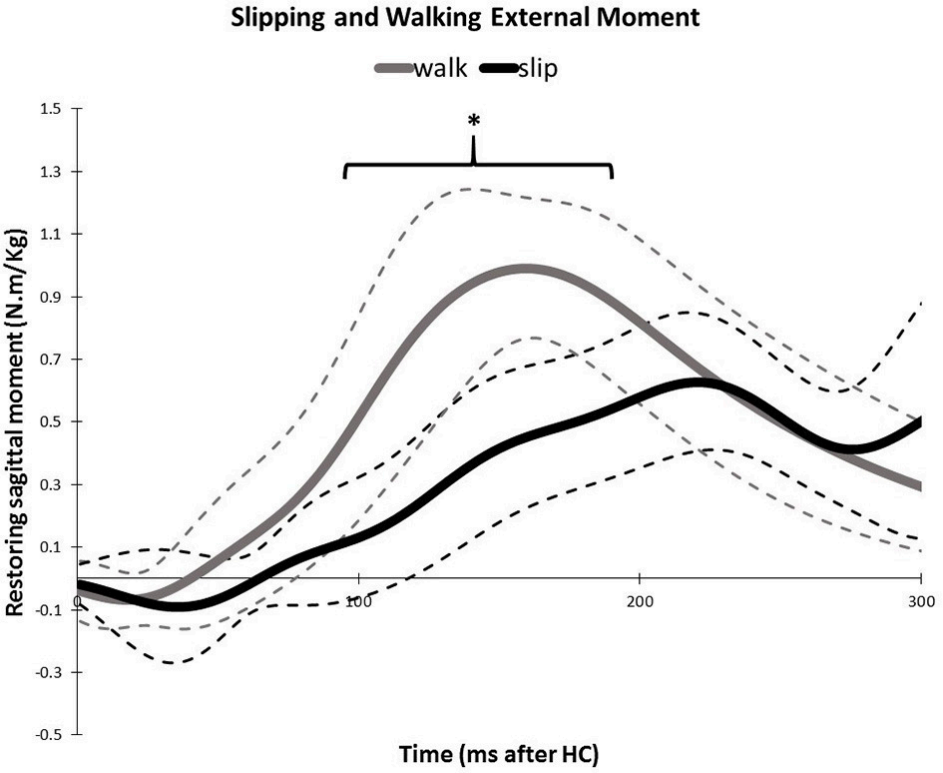
**Average amount of the external mechanical effect (restoring moment) induced on the body after the heel strike for slipping and walking**. Dashed lines indicate one standard deviation. The asterisks indicate statistically significant differences (*p* < 0.05).

Similarity of activation coefficients of the shared synergies for both conditions (Figures 8B, 9B, before 200 ms) agrees with the hypothesis of having the same activation level for the shared synergies before the corrective motor response to slip. This result seems cogent since before the reaction of the body to slip, normal walking and slipping should be dealt with identically and should have the same muscle activation patterns. As a result, before the reaction to a slip, same control blocks (shared muscle synergies) of these “temporarily same” tasks should be activated with the same activation pattern (same activation coefficients). A question may also arise here: The external mechanical effects of the slip (moments imposed on the body) might not deviate from normal walking moments instantly. Hence, can these observed similarities be interpreted as result of the similar moments during “early slipping” and “walking”? To address this question, we calculated the mechanical effect of each condition (i.e., restoring torque in sagittal plane after the heel contact) on the body right using the shear forces generated by the leading limb. We found that the deviations between walking and slipping moments start well before 300 ms post-heel-strike (Figure 12). Using an independent *t*-test, we also found that the restoring moments are significantly different between slipping and walking conditions from 70 to 202 ms (*p* < 0.05) (Figure 12). Thus, the studied interval (300 ms) encompasses different external mechanical effects of slipping and walking on the body. Consequently, we claim that the observed similarities show that the control blocks used for these motor-tasks (synergies) are common, rather than that the motor-tasks are similar. Characteristics of the extracted activations for slip synergies match with previous studies. The body started to react to the slip after 200 ms (Cham and Redfern, 2001) via activating the appropriate control blocks, indicated by peaks in activation levels of slip synergies (Figures 8B, 9B). Timing of the peaks is in accordance with the known primary and secondary motor response to slip (Cham and Redfern, 2001). The first peaks seen in the slip synergies belong to the third and the first synergies (leg decelerator synergy; Figures 8B, 10B), dorsiflexing ankle, flexing knee, and extending hip of the slipping foot, bringing the slipping leg back near the body, matching with the introduced primary response to slip by Cham and Redfern (2001). The next peak belonged to the second shared synergy, that extends knee and flexes hip of slipping leg (Figure 9B), or the secondary response to a slip according to Cham and Redfern (2001).

These findings stay consistent with the existing literature. For example, in a study by Chvatal and Ting (2013) it was found that a common set of muscle synergies is utilized to achieve task-level goals during perturbed and unperturbed walking and standing. Although, most of the studies that examined eight muscles for walking reported four muscle synergies (Neptune et al., 2009; Clark et al., 2010), Chvatal and Ting found five to eight synergies for unperturbed walking (average six), three of which were shared with perturbed walking synergies. Their results however, do not dispute our findings since that study used 16 lower extremity muscles, all from one leg (unilateral). Since muscle synergy analysis is sensitive to the original dimension of the data set (i.e., number of the studied muscles), a direct comparison of the number of synergies would not be feasible among these studies. Yet, their results substantiate the notion that similar biomechanical demands between perturbed walking and normal walking is likely to result in the CNS recruiting similar muscle synergies for both tasks. Furthermore, this article only examines the first 300 ms after the slipping, which captures only the reactive response of the CNS to slipping. However, Chvatal and Ting (2013) studied a larger time period, enabling them to investigate both reactive and voluntary response to the perturbations. Finally, Chvatal and Ting (2013) perturbed subjects in different directions while walking. However, slipping typically happens in the forward direction, which complicates the comparison of these studies. Needless to say, both studies reported that perturbed walking would evoke similar motor patterns to those of unperturbed walking.

A muscle synergy approach is significant as it potentially could establish a basis for a more direct motor rehabilitation (Roh et al., 2013). Having the slipping muscle synergies of healthy individuals as a reference, identification of the impaired synergies in patients would be facilitated. Consequently, one could design appropriate therapies and trainings, conducted toward the damaged synergy (sub-task) to reestablish it in order to perform the required phasic mechanical goals and sub-functions.

Limitation of this study was that there was no classification performed on subjects based on their slipping severity. It is probable for subjects to choose different strategies while countering slips with different severity. Thus, classifying the subjects based on their slip severity would be legitimate. However, the number of individuals in each group could prove insufficient for a cogent statistical analysis; preventing further groupings. In the future studies, we would study subjects' slipping synergies for larger number of classified (based on the severity of slips) groups. By doing so, a conclusion could be made whether the discrepancies in the muscle synergies are or are not significant among groups with different slipping severity and how slipping synergies would help diagnosing the possible cause of severe slips. Another interesting aspect would be studying the modifications of the synergies with repeated perturbed trials to look for possible evolutions in synergies and slipping strategies (Ison and Artemiadis, 2015).

## Conclusion

This study extracted muscle synergies for normal walking and slipping among young healthy subjects. We found two shared and two task-specific muscle synergies among eight lower limb muscles for these two tasks. The activation levels for the shared synergies were identical before the onset of the motor response to slip. Also, the sub-tasks executed by the synergies matched with the known sub-tasks of the gait and slip. The significance of our approach in studying slip, is the identification of the synergies used during this motor task. This identification would form a foundation for a novel diagnosis and rehabilitation method, based on the impaired synergies of motor tasks. Future works will include investigation of the inter-subject deviations and discrepancies of slip synergies and its correlation with the severity of slips, which lead to understanding of the factors causing sever slips to happen.

## Author contributions

All of the authors contributed in a fair and equal manner throughout the process of experimenting, analyzing and publishing this article. PH and KB designed and executed the experiment. MN and HY took the significant share of the data analysis, however, PH and KB also contributed. PH, KB, HY, and MN all participated in drafting the manuscript.

### Conflict of interest statement

The authors declare that the research was conducted in the absence of any commercial or financial relationships that could be construed as a potential conflict of interest. The reviewer TK and handling Editor declared their shared affiliation, and the handling Editor states that the process nevertheless met the standards of a fair and objective review.

## Abbreviations

CNS: central nervous system
MH: medial hamstring
TA: tibialis anterior
RF: rectus femoris
MG: medial gastrocnemius
NS: non-slipping (limb)
S: slipping (limb).
